# Mechanism and target treatment of primary immunodeficiency diseases with systemic lupus erythematosus‐like phenotype

**DOI:** 10.1002/pdi3.67

**Published:** 2024-05-27

**Authors:** Shan Liu, Zhiyong Zhang, Xuemei Tang, Xiaodong Zhao, Yunfei An

**Affiliations:** ^1^ Department of Rheumatology and Immunology Children's Hospital of Chongqing Medical University Chongqing China; ^2^ Chongqing Key Laboratory of Child Infection and Immunity Chongqing China; ^3^ National Clinical Research Center for Child Health and Disorders Chongqing China; ^4^ Ministry of Education Key Laboratory of Child Development and Disorders Chongqing China

**Keywords:** inborn errors of immunity (IEI), primary immunodeficiency diseases (PIDs), systemic lupus erythematosus (SLE)

## Abstract

Primary immunodeficiency diseases (PIDs) present a heterogeneous group of diseases with aberrant immune response caused by monogenic mutations. Due to the immune dysfunction and dysregulation, PIDs have a wide clinical spectrum such as infections, autoimmunity, autoinflammation, allergy, and malignancies. Systemic lupus erythematosus (SLE) is a systemic autoimmune disease characterized with multiple autoantibodies and multiple organ damage, which could be the predominant phenotype in patients with PIDs. In recent years, the increasing identification of monogenic causes of SLE and PIDs discloses the partially shared genetic background and common pathogenic process. The study of PIDs with SLE‐like phenotype paves the way for the exploration of lupus pathogenesis and new perspectives in targeted therapies concurrently.

## 1 INTRODUCTION

Primary immunodeficiency diseases (PIDs), also known as inborn errors of immunity, are genetically heterogeneous monogenic diseases, affecting development, maturation, or function of cells essential for immune system. Due to the monogenic nature, PIDs serving as natural gene knock‐out/in models have been received increasing attention. To date, 485 known genetic defects are listed in the latest update of the International Union of Immunological Societies Expert.^1^ In the clinical setting, PIDs present with diverse clinical manifestations, particularly recurrent and refractory infections. Of note, autoimmunity as the second most prevalent phenotype shouldn't be underestimated.

Systemic lupus erythematosus (SLE) is a multisystem autoimmunity disease with variable clinical and laboratory features. The intricate interactions among gene, environment, and immune system contribute to the immunopathogenesis of SLE, resulting in the tissue inflammation and various organs damage.^2^ More recently, genome‐wide association studies (GWASs) provide an opportunity to identify the genetic risk factors of SLE, and more than 100 risk loci have been demonstrated.^3^ Interestingly, there is overlap between genes that confer susceptibility to SLE and those responsible for PIDs. In some PIDs cases, SLE is the initial phenotype and/or the prominent phenotype. Compared with the classic SLE, SLE in PIDs is more severe and is refractory to classical treatment, which is a particular concern for the management of PIDs.

In the present review, we discuss the mechanisms behind the development of SLE‐like phenotype in primary immunodeficiency diseases from the sight of gene background. Further, we summarize the treatment strategy relieving lupus symptoms, exploring the potential treatment based on the mechanism.

## 2 COMPLEMENT DEFICIENCIES

As one of the first lines of host defense, activated complement components mark the pathological materials or transformed/damaged biological materials and then provide the removal signals to phagocytes of the innate immune system.^4^ In parallel, it enhances antibody response and regulates both B‐lymphocyte and T‐lymphocyte activity. Thus, complement system is a functional bridge between innate and adaptive immunity. The relationship between complement system and SLE appears paradoxical. On the one hand, low level complement proteins in activate SLE suggests that consumption and/or deposition of complement components contributes to inflammation reaction, leading to tissue and organ damage. On the other hand, inherited complement deficiencies predisposed to lupus raise the hypothesis that deficiency of complement system is a risk factor for SLE.^5^

The prevalence of SLE phenotype shows significant variations in the order of complement activation cascade. 93% of patients with C1q deficiency develope SLE, more than 70% of whom present with mucocutaneous manifestations and positive ANA.^6^ And followed, SLE can be seen in 57% with C1r/C1s deficiency, 75% with C4 deficiency, and 10% with C2 deficiency.^7^, ^8^ In addition, defects in the lectin pathway such as mannan‐binding lectin–associated serine protease 2 can also contribute to the pathogenesis of SLE.^9^ The pathogenetic mechanisms of early component complement deficiencies with SLE‐like phenotype might be implicated in impaired scavenging of autoantigens, compromised immune tolerance to self‐antigens, defective autoantibodies and immune complex removal, and dysregulated cytokine production.^8^, ^10^

SLE associated with complement deficiency is often treated by the standard therapeutic regimens used in patients with sufficient complements, but only high dosage prednisolone is effective and the symptoms flare up once the dosage reduces.^11^ Of note, manipulation of the complement system by supplementation is proposed as a potential treatment. Complement‐repletion using fresh frozen plasma (FFP) has been used. Regular FFP infusions release the condition of these patients, which means that temporarily restoring complement activity is sufficient to prevent the production of intracellular self‐antigen and maintain homeostasis in immune system. FFP has been reported in SLE with C1q deficiency for 10‐year, demonstrating the safety and effectiveness of this reagent.^12^ Furthermore, C1q is predominantly synthesized in bone marrow, hence bone marrow transplantation would be used as a cure for C1q deficiency.^13^

## 3 TYPE I INTERFERONOPATHIES

Recognition of pathogen‐derived nucleic acids by pattern‐recognition receptors (PRRs) is the first step against microbial threats for immune defense, which induces the generation of inflammatory signaling molecules, such as Type I IFNs.^14^, ^15^ Subsequently, type I IFNs receptor (IFNAR) interacts with the type I IFNs and activates the receptor‐associated protein tyrosine kinases Janus kinase 1 (JAK1) and tyrosine kinase 2 (TYK2), which ultimately initiates the transcription of interferon‐stimulated genes (ISGs) through the phosphorylation of latent cytoplasmic transcription factors signal transducer and activator of transcription (STAT). Nucleic acid sensors are not limited in pathogen‐derived nucleic acids. The recognition of self‐DNA or RNA in the cytosol also initiates a Type I IFNs response, leading to the development of autoimmunity.^16^ In 1979, increased IFNs had been reported in patients of SLE.^17^ Henceforth, studies have repeatedly measured the up‐regulated type I IFN signaling in SLE, which suggests the close association between them.^18^, ^19^, ^20^, ^21^

Type I interferonopathies are a genetically and phenotypically heterogeneous group of monogenic diseases caused by aberrant upregulation of type I IFNs. Based on the gene background, four putative cellular mechanisms at least have been proposed, which add to our understanding of SLE. First, nuclease deficiency leads to endogenous nucleic acid accumulation, enhancing sustained IFN response inappropriately. Second, gene variants that result in enhanced sensitivity or constitutive activation of nucleic acid sensors activate the IFN signal pathway below the threshold or bypass the need for ligand. Third, mutations in extracellular nucleases increase TLR‐dependent IFN responses. Finally, defects in molecules or pathways that negatively regulate type I IFN signaling lead to upregulation of IFN signaling or overproduction of IFNs.^22^ Below, the key points about them are discussed specifically.

### 3.1 AGS and metabolism of intracellular nucleic acids

*TREX1* (AGS1) encodes the cytosolic exonuclease‐ 3′‐DNA repair exonuclease 1 (TREX1), maintaining a DNA‐free space in the cytosol by the rapid degradation of ssDNA and dsDNA.^23^, ^24^ *RNASEH2A* (AGS4), *RNASEH2B* (AGS2) and *RNASEH2C* (AGS3) encode the three subunits of Ribonuclease H2, which can function as a heterotrimeric enzyme to prevent DNA damage by degrading the RNA within an RNA:DNA hybrid and removing ribonucleotides from genomic DNA.^25^ Sterile alpha motif and histidine–aspartic acid domain‐containing protein 1 (SAMHD1) acts as a dGTP‐dependent triphosphohydrolase to maintain genome stability by regrating dNTP pools during DNA replication.^26^ It also promotes clearance of nascent DNA at stalled replication forks.^27^ AGS5 is caused by *SAMHD1* loss‐of‐function mutation.^28^ These mutations cause the improper production and clearance of unneeded DNA (include nucleic acid from the replication of reverse transcription), which impinges on the cGAS‐STING pathway of cytosolic DNA sensing and drive a chronic type I IFN signature and inflammatory response eventually.^24^, ^29^ ADAR1 (adenosine deaminase acting on RNA 1) is an RNA‐editing enzyme that catalyzes adenosine to inosine in dsRNA. Mutation in *ADAR1* (AGS6) destabilizes double‐stranded RNA (dsRNA), enhancing the recognition of endogenous dsRNA by nucleic acid sensors.^30^ *IFIH1* (AGS7) encodes melanoma differentiation‐associated gene 5 (MDA5), and its gain of function induces the constitutive activation nucleic acid sensors. Upon activation of MDA5 by dsRNA binding, the adaptor mitochondrial antiviral signaling protein (MAVS) triggers the transcriptional induction of the genes encoding type I IFNs.^31^

There is another non‐AGS enzyme that illustrates the contribution of metabolism of intracellular nucleic acids as a key mechanism to develop SLE. DNase II is a lysosomal endonuclease. It plays a critical role in the degradation of endocytosed apoptotic cell‐derived DNA. Like TREX1 deficiency, absence of DNase II triggers IFN production through the activation of cGAS‐STING pathway, resulting in the development of SLE.^32^ Of note, all the patients with *DNASE2* mutations were characterized with high titers of anti‐DNA autoantibodies and renal disorders.^33^ In the patient reported recently, neurological changes such as cognitive impairment and learning difficulties were uncovered. Therefore, The role of *DNASE2* in SLE pathogenesis requires further analysis.

### 3.2 Hypersensitive or constitutively activation of nucleic acid sensors

Mutations associated with the nucleic acid sensors or key adaptor proteins upregulate IFNs signaling in the absence of ligand and/or enhanced sensitivity to lower amounts of ligand. As described above, *IFIH1* gain‐of‐mutation belongs to this class of human interferonopathies. Besides, STING‐associated vasculopathy infantile‐onset (SAVI) caused by dominant activating mutations in *TMEM173* is included. *TMEM173* encodes the protein STING, which functions as an adaptor for cytosolic DNA sensing. Activating transcription of type 1 IFN genes and IFN‐response genes in *TMEM173* mutations dependents on the dimerization and constitution activation of STING without cGAMP ligand.^34^ As a result, endothelial‐cell dysfunction located in vessels including inflammatory and vaso‐occlusive process is induced. Mutations in STING is associated with SLE‐like futures.^35^ But rashes in SAVI are aggravated by cold exposure, and the autoantibody titers do not correlate with disease activity.^36^

### 3.3 Metabolism of extracellular nucleic acids and TLR‐dependent IFNs response

DNase1 and DNase1L3 are known to be present in blood. Therefore, cell‐free DNA (cfDNA) derived from apoptosis, necrosis, NETosis and active secretion processes is cleared by them predominantly. Mutations in these DNases break the balance of cfDNA pool, resulting in the activation of autoimmunity response. Unlike the first two classes interferonopathies, mutations in these DNases cause diseases resembling SLE through the activation of the nucleic acid‐sensing TLRs.^22^ The decreased DNASE1 activity and high antibody against nucleosomal antigens demonstrated direct contribution of *DNASE1* loss of function mutations to the progression of human SLE.^37^ DNase1L3 is resemble to DNase1, but DNase1L3 is more causative to SLE. At present, 29 DNase1L3‐deficient patients with SLE had been published.^38^, ^39^, ^40^, ^41^, ^42^, ^43^, ^44^, ^45^ Most patients developed SLE before 6 years old and had obvious presentation with lupus nephritis, recurrent urticarial rash and positive anti‐neutrophil cytoplasmic antibodies. This phenomenon might be attributed to the higher efficiency of DNase1L3 in digestion of larger and multinucleosomal sized cfDNA.^46^, ^47^ However, this cfDNA is more likely to trigger an immune response.

Spondyloenchondrodysplasia (SPENCD) caused by biallelic mutations of *ACP5* is a rare skeletal dysplasia characterized with neurological impairment and immune dysfunction. *ACP5* encodes tartrate resistant acid phosphatase (TRAP), an osteoclast‐derived cytokine that inactivates osteopontin (OPN) by dephosphorylation.^48^ The loss of regulation of TRAP activity leads to the functional excess of phosphorylated OPN, and is followed by increased TLR9‐dependent IFN‐α production of plasmacytoid dendritic cells.^49^, ^50^ Large spectrum of immunologic abnormalities including SLE may be seen in SPENCD. Notably, there is a dramatic up‐regulation of type I interferon especially in these patients, which strongly supports the pathogenesis of SLE phenotype developed by the *APC* variants.^51^

### 3.4 Aberrant regulator of type I IFN signaling

As a member of the ubiquitin family, interferon‐stimulated gene 15 (ISG15) modifies many intracellular substrates via ISGylation, which negatively regulates the type I IFN over‐amplification through the stabilization of ubiquitin‐specific peptidase 18 (USP18) levels.^52^, ^53^ USP18 as a negative–feedback regulator competes with JAK1 recruiting to IFNAR2.^54^ ISG15 deficiency results in no synthesis of USP18, leading to unrestrained IFN–I signaling. Patients with these mutations show SLE clinical manifestations especially cutaneous lesions and abnormal immunological tests.^55^ These patients can be treated with corticosteroids, immunosuppressive drugs and intravenous immunoglobulin. Besides, belimumab is associated with favorable responses to the control of active lupus symptoms.^55^

Since there has been no approved drug in management of SLE phenotype in Type I Interferonopathies, patients are commonly treated according to guideline of the classical SLE. As an immunomodulator, hydroxychloroquine (HCQ) is widely used in SLE. It is surprising that HCQ exerts an anti‐IFN‐α effect by inhibiting dsDNA stimulation of cGAS.^56^, ^57^ Therefore, HCQ can be useful for type I interferonopathies (including TREX1, RNASEH2A‐C and SAMHD1 deficiency) caused by the cGAS‐dependent DNA sensing pathway.^58^, ^59^, ^60^ Nevertheless, it stands to reason that agent selectively interfere with the molecular pathways is more effective than the broadly suppress the immune system. Thus, new and effective drugs are needed urgently for SLE. According the mechanisms involved in Type I Interferonopathies, different strategies are pointed out. Theoretically, therapeutic approaches in the type I interferonopathies might include blocking the generation, sensing and signaling of self‐nucleic acids, the IFNAR receptor and the signaling cascades distal to interferon ligand binding. Case reports of JAK inhibitors in type I interferonopathies such as TREX1 deficiency, AGS and SAVI have been reported.^61^, ^62^, ^63^, ^64^, ^65^, ^66^ Furthermore, based on the increased DNA derived from endogenous retroelements, clinical trial in AGS is conducting via the usage of reverse transcription inhibitors.

## 4 JAK‐STAT SIGNAL PATHWAY

JAK‐STAT signal pathway activated by a wide range of cytokines and growth factors. It plays a vital role in various physiological processes including innate and adaptive immune responses, hematopoiesis, growth, and development.^67^ At present, SLE have been reported in patients with STAT1 gain of function and STAT3 loss of function, which is considered to a consequence of increased amplified transcription of IFN responsive genes.^32^, ^68^

Patients with autosomal dominant gain of function STAT1 mutations impair the dephosphorylation of activated STAT1 protein, causing the accumulation of phosphorylated STAT1 and followed by higher levels of STAT1 responsiveness to IFN.^69^ In addition, hypotheses about IL‐27 through STAT1 have been reported to explain the molecular mechanism of developing SLE manifestations.^70^ As a result, a wide spectrum of clinical manifestations of SLE, including arthritis, serositis, hemolytic anemia, autoimmune thrombocytopenia, proteinuria and neurological disorders, were showed.^32^, ^71^

Based on the treatments in classical SLE, systemic corticosteroids was used among these patients, and lupus symptoms were partially improved.^71^ But clinical disease frequently worse progressively due to the gene variant context. As reported, the oral JAK inhibitor was used in patients with GOF mutation, and normalized both STAT1 protein and pSTAT1 levels.^72^ However, JAK inhibitor in patients with STAT1 gain of function mutation developing SLE phenotype has not been reported. More investigations are needed to demonstrate the safety and availability of the drugs. HSCT seems to be an alternative and curative therapeutic option. But complications including secondary graft failure, infections and bleeding limit its usage and the overall survival was reported as 40%.^73^, ^74^ The secondary graft failure might be related to the enhanced interferon signaling of resident cells in hematological system and tissues, which results in the uncontrolled inflammation.^74^ Recently, ruxolitinib (a JAK inhibitor) acted as a conditioning regimen provides a better outcome after transplantation, which offered better disease management for STAT1 gain of mutation.^75^

Interestingly, several early studies demonstrated that the level of STAT3 links to SLE disease activity closely.^76^, ^77^ STAT3 gain of function often accompanies autoimmunity and autoinflammation.^78^ Thus, STAT3 inhibition is considered to be a target therapy in SLE. However, SLE have also been observed in patients with STAT3 loss of function mutation, suggesting the multiple and distinct biological roles of STAT3 in autoimmunity.^79^ It is reported that STAT3 loss‐of‐function mutations have the clinical features observed in SLE, such as nephritis, autoimmune cytopenia, discoid rash, and alopecia. The probable pathogenesis of SLE in these variants is associated with the impaired induction of suppressor of cytokine signaling 3 (SOCS3), resulting in the upregulation of the STAT1 pathway and followed the increased expression of ISGs.^80^

SLE phenotype in STAT3 loss of function is treated with a combination of immunosuppressants. Surprisingly, these drugs can lead to disease remission. Given the increased expression of ISGs and NET formation, target treatment such as JAK inhibition can be used for SLE symptoms of these mutations.^79^

## 5 TREG DYSFUNCTION

To avoid the autoreactive response, T cells are selected in the thymus. But the recognition of various pathogens provide a random repertoire of antigen receptors for T cells, resulting the presence of autoreactive T cells. In normal immune system, Tregs maintain the immunotolerance state by a key receptor named Cytotoxic T‐lymphocyte protein 4 (CTLA‐4), which can regulate the activity of antigen presenting cells (APC) and naïve T cells through the competence with CD28.^81^ Lipopolysaccharide‐responsive and beige‐like anchor protein (LRBA) controls intracellular trafficking of Cytotoxic T‐lymphocyte protein 4 (CTLA‐4), and a loss of LRBA causes the insufficient CTLA‐4 expression on the surface of Tregs, resulting the development of autoimmune diseases. LRBA deficiency is identified in a SLE patient with polyarthritis, pericarditis, autoimmune hemolytic anemia, alopecia, persistent malar rash and aberrant immunological testing.^82^ According to the function of CTLA‐4, CTLA4‐Ig (abatacept) therapy may be considered in patient with LRBA mutation.

DOCK8 protein is involved in cells signaling about the arrangement of the cytoskeleton, which maintains cells structure and the right location. Therefore, it is critical for the survival and function of immune system cell.^83^ SLE manifestations have been reported in several patients with DOCK8 deficiency.^83^, ^84^, ^85^ The potential pathogenic mechanism is connected with the decreased number and suppressive activity of Tregs binding to the break of peripheral B cell tolerance checkpoint, which was strongly proved by the identification of enriched autoreactive B cells in the mature naïve B cell compartment among DOCK8‐deficient patients.^86^ DOCK8 gene mutation patients with SLE respond to immunosuppressive therapy, but 10‐year follow‐up of a DOCK8‐deficient child with SLE feature reveals more effective therapies are needed.^85^ Base on the availability in various autoimmune diseases (including SLE) and DOCK8 deficiency, HSCT is regarded as a fundamental treatment for these patients.^87^ Normal complement fractions at 1 month post‐HSCT and disappeared autoimmune features at 12 months post‐HSCT highlight the usefulness and curability of HSCT.^83^

## 6 ABERRANT T‐CELL RECEPTOR (TCR) SIGNALING

Phosphoinositide 3‐kinase delta (PI3Kδ) composed of catalytic subunit p110δ and regulatory subunit p85α (encoded by genes *PIK3CD* and *PIK3R1* respectively) is a member of the class IA family of PI3Ks. It transduces signal from cell‐surface receptors by the conversion of phosphatidylinositol 3,4,5‐trisphosphate (PIP_3_), promoting phosphorylation of RACα serine/threonine‐protein kinase (AKT) and activation of downstream proteins involving mammalian target of rapamycin (mTOR).^88^, ^89^ P110δ protein is predominantly expressed in leukocyte, which plays a critical role in B lymphocyte and T lymphocyte development and responses.^90^ Dominant gain‐of‐function mutations in the *PIK3CD* gene or dominant loss‐of‐function mutations in the *PIK3R1* gene cause activated PI3Kδ syndrome, a novel PID also named APDS.^91^ The hyperactivation of PI3Kδ exhibits enhanced AKT‐mTOR signaling, resulting in the expansion and survival of follicular helper T (Tfh) cell and incompetent B cells.^88^ Wide range of autoimmunity manifestations can be seen in patients with APDS, and SLE is a newly phenotype descripted in 2019.^92^, ^93^, ^94^, ^95^ Cytopenias have a benefit in the treatment of steroids.^96^ And given the genetic etiology of this disease, PI3K pathway inhibitors functioning as putative immunosuppressive drugs, may be the effective therapy. Rapamycin, a mTOR inhibitor, has been found to improve symptoms including pericardial effusion, abdominal effusion and proteinuria. But there was no change in positive ANA and low level of C3.^97^ Leniolisib (CDZ173) as a selective oral inhibitor of p110δ can diminish the increased T‐cell senescence in APDS, which indicates that it might be a new treatment option for these patients.^98^ Although these targeted therapies appears attractive, serious side effects reported can't be ignored. Therefore, more clinical trials should be carried out to titrate the best dose and weight the long‐term risks and benefits.

Purine nucleoside phosphorylase (PNP) is a critical enzyme in the purine salvage pathway. PNP deficiency leads to the generation of deoxyguanosine triphosphate (dGTP). As a lymph‐toxic metabolite, dGTP inhibits ribonucleotide reductase and impairs T‐cell maturation and differentiation consequently. Due to the protective effect of high nucleotidase activity, B cell number and function can be normal.^99^ PNP deficiency has variable clinical presentations including infection, neurological impairment and autoimmunity. It is reported that autoimmune complications exist in one‐third of patients with this disease. And SLE phenotype is found to have relation with PNP deficiency from 1991. At present, there are three patients with this disease who also have SLE, which may be attributed to the immune dysregulation and disturbed of the cellular homeostasis in T cells.^32^, ^100^, ^101^ In consideration of severe infection, HSCT is curative treatment for PNP deficiency.^102^

## 7 B‐CELL‐INTRINSIC DEFECTS

As an essential component of a signaling pathway, PKCδ induces the homeostasis and tolerance in B cells. PKCδ protein is encoded by gene *PRKCD*. Biallelic loss‐of‐function mutations in *PRKCD* reduce expression and activity of PKCδ, resulting in B‐cell hyperproliferation and defective apoptosis.^103^ Since SLE is predominantly caused by loss of immune tolerance to autoantigens, the impairment of the B cell tolerance checkpoint in PKCδ deficiency contributes to the development of lupus phenotype. To date, at least six patients with PKCδ deficiency from four unrelated families have been reported to present with SLE phenotype.^104^, ^105^, ^106^, ^107^ All of these patients developed SLE before the age of 10 years, and manifestations such as lupus rash, lupus nephritis and arthritis are involved. Based on the nature of this disease, B cell depletion is likely to have a logical targeted therapeutic role for these patients. Rituximab as a chimeric monoclonal antibody that targets CD20 positive B cells improves clinical outcomes.^108^ But severe infusion reactions is its main limitation.^104^ Ofatumumab is a fully humanized monoclonal antibody that targets B cells from a different epitope of CD20. Patients intolerant of rituximab showed a good response to ofatumumab.^104^ Interestingly, hydroxychloroquine as a classical SLE treatment improved skin feature and lymphoproliferation in patient with PKCδ deficiency, which revealed that autoreactive B cells could be modulated by hydroxychloroquine.^109^

## 8 APOPTOSIS DEFECTS

As prototypical members of the small GTPases, Ras proteins induce a wide range of cellular processes, such as cell proliferation, differentiation, survival, apoptosis and gene expression.^110^ In addition, *NRAS* mutation and *KRAS* mutation are responsible for the non‐malignant lymphoproliferation disease called Ras‐associated autoimmune leukoproliferative disorder (RALD). Since its first identification, RALD was designated as the type IV of autoimmune lymphoproliferative syndrome (ALPS).^111^ Previous study indicated that patients with this disease may have elevated or normal TCRαβ+ CD4‐ CD8‐ lymphocyte.^112^, ^113^ Given that the increased TCRαβ+ CD4‐ CD8‐ lymphocyte is one of the most specific findings for APLS, a new nomenclature of RALD was used in 2019.^114^ Manifestations including severe hematologic changes, arthritis, low level of complements were observed in RALD patients, which were often identified as SLE at first.^115^, ^116^ NRAS and KRAS proteins have found to suppress the proapoptotic protein BCL‐2 interacting mediator of cell death (BIM), leading to impaired intrinsic T‐cell apoptosis.^113^ Therefore, the SLE phenotype in RALD can be attributed to defects in lymphocyte apoptosis. In view of precise medicine, small molecule inhibitors of kinase effectors of Ras‐GTP are the optimal treatment for RALD. But it is disappointing that these drugs are mainly used as anticancer agents.^117^ Fortunately, most of SLE symptoms is released by corticosteroid and/or immunosuppressive drugs.

## 9 CONGENITAL DEFECTS OF PHAGOCYTE FUNCTION

Reactive oxygen species (ROS) generated mainly by the activation of nicotinamide adenine dinucleotide phosphate (NADPH) oxidase (NOX) complex during respiratory burst are essential for antimicrobial and immunoregulatory function of Phagocytic cell.^118^ Mutations in genes (such as *CYBB*, *NCF1*, *NCF2* and *NCF4*) encoding the subunits of NADPH oxidase complex lead to chronic granulomatous disease (CGD), which is charactered by severe infection (particularly catalase‐positive bacteria and fungi) and tissue granuloma formation due to the low production of ROS.^119^ Besides, CGD has an increased risk of developing autoimmune diseases like SLE. Discoid lupus was noted both in carriers and in patients with CGD at first.^120^, ^121^, ^122^, ^123^, ^124^ Subsequently, several studies reported that CGD could manifest with SLE symptoms including photosensitivity, malar rash, glomerulonephritis, leukopenia, hypocomplementemia, ANA and dsDNA.^125^, ^126^, ^127^ The possible mechanisms proposed by the researchers are listed as follows: Impaired phagocytosis leads to the overburden of biological waste and the exposure of autoantigens.^128^ And decreased information of neutrophil extracellular traps (NETs) prevents the proinflammatory material from degradation, resulting in unlimited inflammation.^129^ Type I IFN response is contributed to oxidized mitochondrial DNA generated by increased mitochondrial ROS and enhanced JAK/STAT pathway.^130^, ^131^ In addition, low ROS production enhances the presentation of autoantigens in macrophage.^132^ Therefore, SLE features in CGD appears to have self‐amplifying nature because of a pathogenic interplay among ineffective immune tolerance, hyperactive autoantigens presentation, upregulate JAK/STAT signal and tissue damage. Typical SLE treatment was used in the management of SLE symptoms in CGD. HCQ is effective for discoid lupus, and transplantation is chosen in some patients.^123^ Activator of the NOX2 complex might be the potential treatment in the future.^129^

## 10 CONCLUSION

PIDs composed of 465 known genetic defects are heterogeneous diseases that mainly impair the development and function of immune system, which present a diverse spectrum of presentations. As it has been explored in this review, more than 30 genes responsible for PIDs confer susceptibility to SLE, which leads to critical insights into SLE pathogenesis. Mechanisms involved include complement deficiency, abnormal upregulation of type I IFNs, JAK‐STAT pathway dysfunction, ineffective central and peripheral tolerance, apoptosis defects and low production of ROS. To date, there are only a few patients reported. But as natural pathogenesis models, these patients attract broad attention. And based on these genes background, more effective treatments (see table 1) such as JAK1/2 and mTOR inhibitor are explored. However, due to the limited number of patients, more trials and long‐term follow‐up are needed to evaluate the risks and benefits of the targeted drugs. New drugs such as reverse transcriptase inhibitor, nucleic acid degradation agonists, or even gene therapy need exploring.

**TABLE 1:** Therapeutic options of primary immunodeficiency diseases with systemic lupus erythematosus‐like phenotype.

Mechanism	Disease	Gene	Inheritance	Main therapy
Complement pathway	C1 deficiency	*C1q/r/s*	AR	Manipulation of the complement system (like FFP),^1^ marrow transplantation^2^
	C2 deficiency	*C2*	AR	
	C4 deficiency	*C4*	AR	
	MASP2 deficiency	*MASP2*	AR	
Type I interferon signal	AGS1	*TREX1*	AR/AD	JAK inhibitors,^3^, ^4^, ^5^, ^6^ reverse transcription inhibitors for AGS^7^
	AGS2	*RNASEH2B*	AR	
	AGS3	*RNASEH2C*	AR	
	AGS4	*RNASEH2A*	AR	
	AGS5	*SAMHD1*	AR	
	AGS6	*ADAR1*	AR	
	AGS7	*IFIH1*	AD GOF	
	DNAse II deficiency	*DNASE2*	AR	
	SAVI	*TMEM173*	AD	
	Pediatric systemic lupus erythematosus due to DNASE1L3 deficiency	*DNASE1L3*	AR	
	SPENCD	*ACP5*	AR	
	ISG15 deficiency	*ISG15*	AR	
JAK‐STAT signal pathway	STAT1 GOF	*STAT1*	AD GOF	JAK inhibitor,^89^ HSCT^10^, ^11^
	STAT3 mutation	*STAT3*	AD LOF/GOF	
Treg dysfunction	LRBA deficiency	*LRBA*	AR	CTLA4‐Ig (abatacept) therapy,^12^ HSCT^13^
	DOCK8 deficiency	*DOCK8*	AR	
Aberrant TCR signaling	APDS	*PIK3CD GOF/PIK3R1*	AD	PI3K pathway inhibitors (mTOR inhibitor,^14^ p110δ inhibitor^15^),
	PNP deficiency	*PNP*	AR	HSCT^16^
B‐cell‐intrinsic defects	PRKCD deficiency	*PRKCD*	AR	B Cell depletion (monoclonal antibody that targets B cells)^17^, ^18^
Apoptosis defects	RALD	Somatic mutation in *NRAS/KRAS* (GOF)		Potential strategy‐inhibitors of kinase effectors of Ras‐GTP
Congenital defects of phagocyte function	CGD	*CYBB*	XL	Potential strategy‐activator of the NOX2 complex
		*NCF1/NCF2*	AR	

## AUTHOR CONTRIBUTION

Shan Liu is responsible for writing the manuscript. Zhiyong Zhang is responsible for translating and revising. Xuemei Tang and Xiaodong Zhao are responsible for revising. Yunfei An is responsible for reviewing and revising.

## CONFLICT OF INTEREST STATEMENT

Xiaodong Zhao is the Deputy Editor‐in‐Chief of Pediatric Discovery. To minimize bias, he was excluded from all editorial decision‐making related to the acceptance of this article for publication. The remaining authors declare no conflict of interest.

## ETHICS STATEMENT

Not applicable.

## Data Availability

Data sharing is not applicable to this article as no new data were created or analyzed in this study.
